# Association between blood urea nitrogen to serum albumin ratio and in-hospital mortality of patients with sepsis in intensive care: A retrospective analysis of the fourth-generation Medical Information Mart for Intensive Care database

**DOI:** 10.3389/fnut.2022.967332

**Published:** 2022-11-04

**Authors:** Shaoyan Cai, Qinjia Wang, Chao Chen, Chunming Guo, Liangjie Zheng, Min Yuan

**Affiliations:** ^1^Department of Anesthesiology, Shantou Central Hospital, Shantou, Guangdong, China; ^2^Department of Gastroenterology, First Affiliated Hospital of Shantou University Medical College, Shantou, Guangdong, China; ^3^Department of Neurology, Jiangxi Provincial People’s Hospital, The First Affiliated Hospital of Nanchang Medical College, Nanchang, China

**Keywords:** blood urea nitrogen to serum albumin ratio, sepsis, MIMIC-IV, in-hospital mortality, association

## Abstract

**Background:**

This study aimed to investigate the relationship between the blood urea nitrogen to serum albumin ratio (BAR) and in-hospital mortality in patients with sepsis.

**Materials and methods:**

This is a retrospective cohort study. All septic patient data for the study were obtained from the intensive care unit of Beth Israel Deaconess Medical Center. Adjusted hazard ratios (HRs) and 95% confidence intervals (CIs) were calculated using multivariable Cox regression analyses. Survival curves were plotted and subgroup analyses were stratified by relevant covariates.

**Results:**

Among 23,901 patients, 13,464 with sepsis were included. The overall in-hospital mortality rate was 18.9% (2550/13464). After adjustment for confounding factors, patients in the highest BAR quartile had an increased risk of sepsis death than those in the lowest BAR quartile (HR: 1.42, 95% CI: 1.3–1.55), using BAR as a categorical variable. When BAR was presented as a continuous variable, the prevalence of in-hospital sepsis-related death increased by 8% (adjusted HR: 1.08, 95% CI: 1.07–1.1, *P* < 0.001) for each 5-unit increase in BAR, irrespective of confounders. Stratified analyses indicated age interactions (*P* < 0.001), and the correlation between BAR and the probability of dying due to sepsis was stable.

**Conclusion:**

BAR was significantly associated with in-hospital mortality in intensive care patients with sepsis. A higher BAR in patients with sepsis is associated with a worse prognosis in the ICU in the USA. However, further research is required to confirm this finding.

## Introduction

Sepsis is a serious global public health issue and is often defined as life-threatening organ dysfunction caused by a dysregulated host response to infection (1, 2). Despite advances in the diagnosis and treatment of sepsis (3), sepsis-related deaths account for 19.7% of all deaths worldwide (4–6).

Therefore, clinical physicians should identify patients with potentially high-risk sepsis (7). Using risk profiles or clinical indicators that predict disease severity and prognosis, clinicians can make better management decisions to reduce the incidence of multiple organ failure and relative mortality (8). Hematological and biochemical biomarkers facilitate diagnostic and treatment processes (9). Traditional biomarkers such as procalcitonin (PCT), C-reactive protein (CRP), interleukin (IL)-6, lactate, or white blood cell count, which reflect disease severity, may lack specificity to distinguish the condition (7, 10). Other biomarkers of sepsis, such as lipopolysaccharide-binding protein (LBP), soluble trigger receptor-1 (sTREM-1), and soluble urokinase plasminogen activator receptor (suPAR) expressed on myeloid cells have low sensitivity and specificity (9, 11). The diagnostic and prognostic value of presepsin, a soluble cluster of 14 subtypes (sCD14), in sepsis is controversial (12, 13). Hence, the most credible and reliable biomarker to predict the prognosis of patients with sepsis has not been established yet.

Blood urea nitrogen (BUN) and serum albumin are low-cost biomarkers that are widely used in clinical practice (14, 15). Several studies have identified hypoalbuminemia as an independent risk factor for mortality and poor prognosis after infection (15, 16). BUN is an important parameter that reflects the relationship between renal condition, protein metabolism, and nutritional status (17, 18). A strong association has been reported between BUN levels and mortality due to sepsis (19). The blood urea nitrogen to serum albumin ratio (BAR), which combines nutritional status and renal status, has recently been found to be a valuable tool for predicting mortality in critically ill patients (20). The BAR is thought to be related to the nutritional status of the organism, dehydration status, and liver and kidney functional reserves (21). It is considered a significant predictor of prognosis for different kinds of diseases, including community-acquired pneumonia (22), gastrointestinal bleeding (23), pulmonary embolism (24), and novel coronavirus pneumonia (25). Nevertheless, there are currently few published studies on the relationship between BAR and the prognosis of patients with sepsis, and the former studies had a limited sample size. Consequently, we hypothesized that BAR is a potential independent risk factor for sepsis prognosis. To validate our hypothesis, we used data from the fourth-generation Medical Information Mart for Intensive Care (MIMIC-IV, version 1.0) database to preliminarily investigate the association between BAR and the prognosis of patients with sepsis in the ICU.

## Materials and methods

### Data source

This retrospective cohort study was conducted according to the Strengthening the Reporting of Observational Studies in Epidemiology guidelines (26). We enrolled patients with sepsis from the MIMIC-IV database of the Massachusetts Institute of Technology (MIT). More than 70,000 adult patients were admitted to the ICU of Beth Israel Deaconess Medical Center in Boston between 2008 and 2019. The requirement for informed consent or ethical approval was waived because the data were obtained from publicly available sources (27) with de-identified information. The first author, Shaoyan Cai, completed the training on “Human Subject Protection” and gained full access to the database (certification number 46658933). Raw data were extracted using Structured Query Language with PostgreSQL 13.0 and Navicat Premium 15.

### Participants

According to the MIMIC-IV database, the total number of patients admitted was 76,540 from 2008 to 2019, with 53,150 of them first admitted to the ICU. The inclusion criteria of this study included patients aged >18 years who fulfilled the Sepsis-3 criteria (1). Sepsis-3 is defined as a increase of ≥2 points in the sequential organ failure assessment (SOFA) score plus documented or suspected infection (1, 28). Septic shock was defined by the International Classification of Diseases (ICD) code 78552 (ninth revision) and ICD code R6521 (10th revision) (29, 30). Exclusion criteria included patients aged <18 years, patients whose BUN and albumin levels were not available during the first day of ICU admission, and patients without the length of hospital time. Acute kidney injury (AKI) was defined by Kidney Disease: Improving Global Outcomes (KDIGO) criteria, using both SCr and urine output criteria (31). The lowest SCr values available within 7 days before admission were used as the baseline SCr (32). When SCr data prior to admission were not available, the first SCr measured on ICU admission was used as the baseline SCr (33). For patients admitted multiple times to the ICU, we only considered the data from the first ICU admission (34, 35).

### Variates

Based on previous literature (36–38) and our clinical practice, we chose the following variables considered to be confounding factors for the prognosis of sepsis.

Demographic and admission conditions: age, sex, ethnicity, weight, AKI, malignant cancer, severe liver disease, renal disease, urine output, and severity at admission as measured by the SOFA score, acute physiology score (APS) III, and Charlson comorbidity index (CCI).

Vital signs: first measurements of mean arterial pressure (MAP), heart rate (HR), and oxygen saturation level (SpO_2_) at ICU admission.

Interventions: use of mechanical ventilation, renal replacement treatment (RRT), and vasopressin usage during the first 24 h of ICU admission.

Laboratory results: hemoglobin, SCr, glucose, white blood cell (WBC) count, hemoglobin, platelet count, chloride, lactate, and pH levels.

The BAR was calculated as the quotient of initial blood urea nitrogen (mg/dl) and serum albumin (g/dL) based on laboratory results at ICU admission. If the above parameters had more than one result within 24 h, the first set of data was chosen.

### Outcome

The study outcome was in-hospital mortality, defined as the survival status at hospital discharge. The follow-up time was surveyed from the time of admission to the event of discharge or death. Patients without any recorded outcome or follow-up time were excluded from the final cohort.

### Statistical analysis

Descriptive analysis was performed for categorical variables according to BAR quartiles (<4.85; 4.85–7.86; 7.86–13.9; ≥13.9) using the Kruskal–Wallis test or one-way analysis. For categorical variables, baseline characteristic data were presented as proportions (%) and compared using chi-square tests. Normally distributed continuous data were presented as mean ± standard deviation (SD) and compared using Student’s *t*-test between groups, while skewed distribution median data are presented as the interquartile range (IQR) and compared using the Wilcoxon rank-sum test.

We constructed three multivariate Cox proportional hazards models to assess the independent association between BAR and in-hospital mortality: Model 1 was adjusted only for age and sex; Model 2 was additionally adjusted for ethnicity, MAP, HR, SpO_2_, hemoglobin, SCr, PLT, WBC, chloride, glucose, lactate, and pH levels; and Model 3 was additionally adjusted for weight, malignant cancer, urine output, severe liver disease, renal disease, SOFA score, APS III, urine output, ventilator use, CCI, RRT use, and vasopressin usage. A test for linear trends was conducted using quartiles of the exposure variable as a continuous variable by assigning the median values of the quartiles to the variable.

Stratified and interaction analyses were applied according to sex (male or female), age (<65 or ≥65 years), SOFA score (<5 or ≥5), ventilator use (yes or no), RRT use (yes or no), vasopressin usage (yes or no), septic shock (yes or no), and AKI (yes or no). Each stratification was adjusted for all factors (including age, sex, ethnicity, HR, MAP, SpO_2_, hemoglobin, SCr, platelets, WBC, chloride, glucose, lactate, pH, weight, malignant cancer, severe liver disease, renal disease, CCI, APSIII, SOFA score, urine output, ventilator use, RRT use, and vasopressin usage) except for the stratification factor itself.

Hospital survival was assessed using Kaplan–Meier survival curves according to the BAR quartiles and evaluated using the log-rank test (only patients with a hospital length of stay ≤ 30 days were displayed).

The percentage of covariates with missing data was <30% for all analyses. Missing values of covariates were imputed *via* multiple imputations. We created and analyzed three datasets. To assess the robustness of the findings, we applied sensitivity analysis to patients after excluding participants with missing data (Supplementary Table 1). We excluded patients with ICU stays <24 h and <48 h as a sensitivity analysis (Supplementary Tables 2, 3). Additionally, we applied sensitivity analysis to assess the association between BAR and in-hospital mortality in the population of AKI (Supplementary Table 4).

The data analysis process was performed using packages R3.6.3 (The R Foundation^1^) software and Free Statistics software version 1.5. *P*-values < 0.05 were considered significant.

## Results

### Population

**FIGURE 1:**
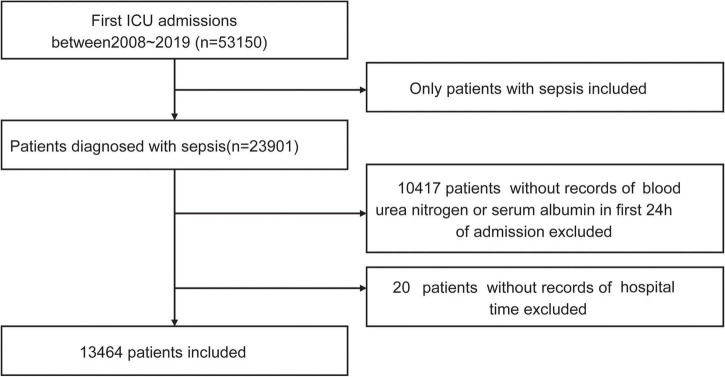
Flowchart of study patients.

A total of 23,901 patients with sepsis were identified. After excluding 10,437 patients without a BAR value or in-hospital time, 13,464 patients with sepsis were included in the final data analysis as shown in the flow chart (Figure 1).

### Baseline characteristics

The basic demographic characteristics of all selected patients stratified by the BAR quartile are shown in Table 1. Overall, the age of all participants was 65.3 ± 16.6 years, and 42.8% of them were female participants. The in-hospital mortality rate was 18.9% (2250/13464). Patients in the highest BAR group (Q4) had higher values for age, weight, WBC, BUN, SCr, BAR, lactate, glucose, APS III, CCI, and SOFA score, as well as a higher representation of medical aid, renal disease, severe liver disease, RRT, vasopressin usage, septic shock, AKI, and death than those in the other groups. Patients in the Q4 had lower values or incidence for covariates of the female sex, urine output, MAP, SpO2, hemoglobin, albumin, chloride, pH, and ventilator use.

**TABLE 1:** Baseline characteristics of participants and outcome parameters.

Variables	All patients	Q1	Q2	Q3	Q4	*P*-value
		(BAR > 4.85)	(4.85 ≤ BAR < 7.86)	(7.86 ≤ BAR < 13.9)	(BAR ≥ 13.9)	
	(*n* = 13,464)	(*n* = 3,351)	(*n* = 3,373)	(*n* = 3,374)	(*n* = 3,366)	
Age (year)	65.3 ± 16.6	56.7 ± 17.3	65.9 ± 15.8	69.0 ± 15.2	69.5 ± 14.6	<0.001
Female (%)	5767 (42.8)	1641 (49)	1399 (41.5)	1418 (42)	1309 (38.9)	<0.001
Ethnicity, white (%)	8681 (64.5)	2017 (60.2)	2244 (66.5)	2223 (65.9)	2197 (65.3)	<0.001
Insurance, Medicaid (%)	6080 (45.2)	1079 (32.2)	1494 (44.3)	1718 (50.9)	1789 (53.1)	<0.001
Weight (kg)	82.7 ± 24.4	80.2 ± 22.3	82.7 ± 23.0	83.3 ± 25.6	84.5 ± 26.1	<0.001
Urine output (ml)	1703.7 ± 1315.4	2248.0 ± 1425.4	1766.6 ± 1283.4	1483.2 ± 1153.3	1301.7 ± 1180.4	<0.001
**Vital signs**						
Heart rate (bpm)	88.6 ± 17.0	88.8 ± 16.4	87.7 ± 16.7	89.0 ± 17.1	89.0 ± 17.6	0.002
MAP (mmHg)	77.0 ± 10.7	80.1 ± 10.7	78.0 ± 10.5	75.8 ± 10.3	74.0 ± 10.5	<0.001
SPO2 (%)	96.8 ± 2.5	97.2 ± 2.0	96.9 ± 2.4	96.7 ± 2.6	96.6 ± 3.0	<0.001
**Score system, points**						
SOFA score	3.9 ± 2.2	3.2 ± 1.5	3.5 ± 1.9	4.0 ± 2.3	4.8 ± 2.7	<0.001
APS III	61.3 ± 26.7	46.8 ± 21.7	55.6 ± 22.9	65.5 ± 25.4	77.3 ± 26.2	<0.001
CCI	6.0 ± 3.0	4.3 ± 2.8	5.7 ± 2.7	6.6 ± 2.8	7.3 ± 2.9	<0.001
**Laboratory results**						
Hemoglobin (g/dL)	10.5 ± 2.0	10.9 ± 1.9	10.8 ± 2.0	10.3 ± 2.0	9.8 ± 2.0	<0.001
Platelet (k/uL)	179.5 (120.5, 249.5)	191.0 (132.0, 259.0)	180.0 (127.5, 245.0)	172.0 (113.5, 242.5)	173.0 (109.0, 252.0)	<0.001
WBC (k/uL)	11.9 (8.4, 16.3)	11.2 (7.9, 15.0)	11.9 (8.6, 16.0)	12.0 (8.3, 16.5)	13.0 (8.7, 18.4)	<0.001
BUN (mg/dl)	30.5 ± 24.0	11.1 ± 3.3	18.7 ± 3.9	29.6 ± 7.1	62.7 ± 26.1	<0.001
Albumin (g/dL)	2.9 ± 0.6	3.2 ± 0.5	3.0 ± 0.5	2.9 ± 0.5	2.7 ± 0.6	<0.001
BAR (mg/g)	11.0 ± 9.4	3.5 ± 0.9	6.3 ± 0.9	10.4 ± 1.7	24.0 ± 10.2	<0.001
SCr (mg/dL)	1.1 (0.8, 1.8)	0.8 (0.6, 0.9)	1.0 (0.8, 1.2)	1.4 (1.0, 1.8)	2.4 (1.6, 3.8)	<0.001
Chloride (mEq/L)	104.0 ± 6.8	104.2 ± 5.8	104.5 ± 5.9	103.8 ± 6.4	103.4 ± 8.5	<0.001
Glucose (mg/dL)	8.4 ± 3.8	7.5 ± 2.7	8.2 ± 3.2	8.7 ± 4.1	9.0 ± 4.6	<0.001
Lactate (g/dl)	2.00 (1.35, 3.20)	1.80 (1.25, 2.70)	2.00 (1.40, 3.10)	2.15 (1.45, 3.55)	2.10 (1.40, 3.50)	<0.001
pH	7.35 ± 0.09	7.38 ± 0.07	7.36 ± 0.08	7.35 ± 0.09	7.33 ± 0.09	<0.001
**Interventions**						
Ventilator use (%)	6690 (49.7)	1720 (51.3)	1776 (52.7)	1671 (49.5)	1523 (45.2)	<0.001
RRT use (%)	853 (6.3)	31 (0.9)	72 (2.1)	239 (7.1)	511 (15.2)	<0.001
Vasopressin usage (%)	1234 (9.2)	116 (3.5)	239 (7.1)	371 (11)	508 (15.1)	<0.001
**Comorbidity disease**						
Renal disease (%)	3025 (22.5)	98 (2.9)	409 (12.1)	991 (29.4)	1527 (45.4)	<0.001
Malignant cancer (%)	2136 (15.9)	454 (13.5)	536 (15.9)	584 (17.3)	562 (16.7)	<0.001
Severe liver disease (%)	1523 (11.3)	296 (8.8)	305 (9)	427 (12.7)	495 (14.7)	<0.001
Septic shock (%)	2916 (21.7)	354 (10.6)	529 (15.7)	818 (24.2)	1215 (36.1)	<0.001
AKI (%)	7667 (56.9)	1304 (38.9)	1866 (55.3)	2173 (64.4)	2324 (69)	<0.001
Death (%)	2550 (18.9)	319 (9.5)	472 (14)	733 (21.7)	1026 (30.5)	<0.001

Data are presented as mean ± SD or median (IQR) for skewed variables or numbers (proportions) for categorical variables. Q1, BAR < 4.85; Q2, 4.85 ≤ BAR < 7.86; Q3, 7.86 ≤ BAR < 13.9; Q4, BAR ≥ 13.9. Bpm, beats per minute; MAP, mean arterial pressure; SOFA score, sequential organ failure assessment score; APS III, acute physiology score III; CCI, Charlson comorbidity index; WBC, white blood count; SCr, serum creatinine; BUN, blood urea nitrogen; BAR, blood urea nitrogen to serum albumin ratio; RRT, renal replacement treatment; AKI, acute kidney injury.

### Multivariable Cox regression analysis

We constructed three multivariate Cox regression models to evaluate the independent impact of BAR on in-hospital mortality (multivariate Cox regression model). Hazard ratios (HRs) and 95% confidence intervals (CIs) are shown in Table 2. The HRs were robust between the unadjusted and adjusted models in all three models (*P* < 0.05). In the unadjusted model, the effect size of BAR for in-hospital mortality means that a difference of 5 units of BAR is associated with an in-hospital mortality difference of 15% (HR: 1.15, 95% CI: 1.14–1.15). In the minimum-adjusted model (Model 1), the effect size of BAR for in-hospital mortality was increased by 5 units and increased by 13% (HR: 1.13, 95% CI: 1.12–1.14). For each additional 5 units of BAR in the fully adjusted model (Model 3) (adjusted covariates of age, sex, ethnicity, weight, MAP, HR, SpO_2_, hemoglobin, PLT, WBC, lactate, pH, SOFA score, APS III, ventilator use, diabetes, CCI, and Vasopressin usage), the effect size increased by 8% (HR: 1.08, 95% CI: 1.07–1.1). For further sensitivity analysis, the continuous variable BAR was converted into a categorical variable (quartile of BAR), of which the first category of BAR was used as a baseline reference. Patients in the highest BAR quartile had increased in-hospital mortality compared to patients in the lowest BAR quartile (HR: 1.42, 95% CI: 1.3–1.55). The *P*-value for trends in the fully adjusted model for BAR as a categorical variable was calculated with the results when BAR was a continuous variable.

**TABLE 2:** Multivariable Cox regression analysis to assess the association between BAR and in-hospital mortality.

Variable	Unadjusted		Model 1		Model 2		Model 3	
	HR 95% CI	*P*-value	HR 95% CI	*P*-value	HR 95% CI	*P*-value	HR 95% CI	*P*-value
BAR^a^	1.15 (1.14∼1.15)	<0.001	1.13 (1.12∼1.14)	<0.001	1.14 (1.13∼1.16)	<0.001	1.08 (1.07∼1.1)	<0.001
**BAR4**								
Q1 (BAR < 4.85)	1 (Ref)		1 (Ref)		1 (Ref)		1 (Ref)	
Q2 (4.85 ≤ BAR < 7.86)	1.42 (1.3∼1.54)	<0.001	1.28 (1.18∼1.39)	<0.001	1.12 (1.03∼1.22)	0.008	1 (0.92∼1.08)	0.941
Q3 (7.86 ≤ BAR < 13.9)	2.19 (2.03∼2.37)	<0.001	1.91 (1.76∼2.06)	<0.001	1.43 (1.32∼1.55)	<0.001	1.11 (1.02∼1.2)	0.016
Q4 (BAR ≥ 13.9)	3.03 (2.81∼3.25)	<0.001	2.64 (2.45∼2.85)	<0.001	2.07 (1.9∼2.26)	<0.001	1.42 (1.3∼1.55)	<0.001
*P* for trend		<0.001		<0.001		<0.001		<0.001

BAR, Blood urea nitrogen to serum albumin ratio. ^a^BAR was entered as a continuous variable per 5 unit. Model 1 = Adjusted for (age + gender). Model 2 = Model1 + (ethnicity + HR + MAP + SpO_2_ + hemoglobin + SCr + platelets + WBC + chloride + glucose + lactate + pH). Model 3 = Model 2 + (weight + malignant cancer + severe liver disease + renal disease + CCI + APSIII + SOFA score + urine output + ventilator use + RRT use + vasopressin usage).

#### Kaplan–Meier curves

**FIGURE 2:**
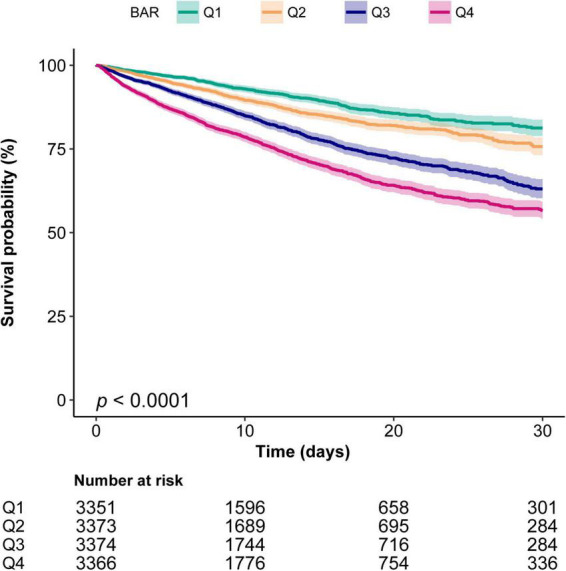
Kaplan–Meier curves indicates the association between the BAR and in-hospital mortality of patients with sepsis. Q1, BAR < 4.85; Q2, 4.85 ≤ BAR < 7.86; Q3, 7.86 ≤ BAR < 13.9; Q4, BAR ≥ 13.9. The curved line and shaded areas depict the estimated values and their corresponding 95% confidence intervals. Only patients with a hospital length of stay ≤ 30 days are displayed.

The Kaplan–Meier curve demonstrated that the in-hospital survival of the highest BAR quantile (Q4) patients was the lowest of all groups, which declined with declining baseline BAR (log-rank test: *P* < 0.0001, Figure 2).

#### Subgroup analysis

**FIGURE 3:**
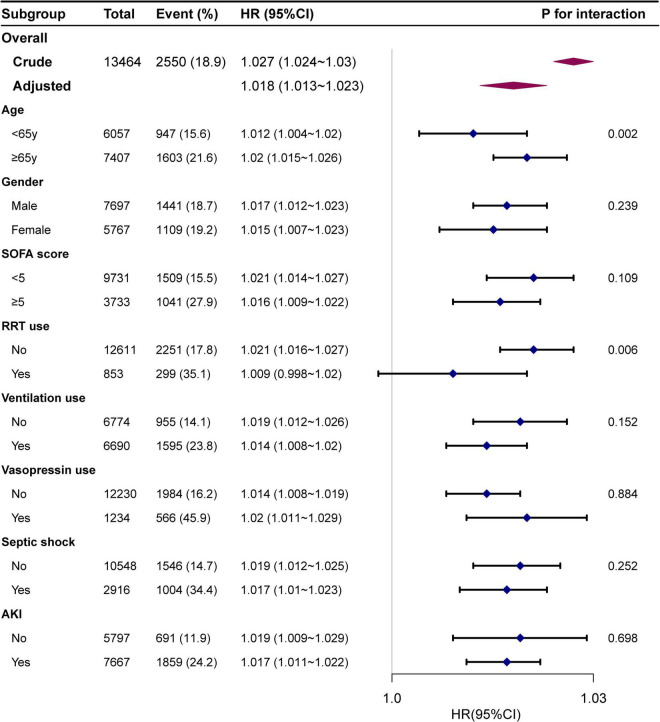
Association between BAR and in-hospital mortality according to baseline characteristics. Each stratification was adjusted for all factors of Model 3 in Table 2 except for the stratification factor itself.

The interaction analysis indicated that BAR was associated with a high risk of ICU mortality in patients aged ≥ 65 years and those using RRT. There were age interactions (*P* = 0.002 for the interaction likelihood ratio test) between BAR and the risk of in-hospital mortality events. As the BAR increased, the in-hospital mortality in the elderly subgroup (age ≥ 65 years) significantly increased (HR: 1.025; 95% CI: 1.019–1.032). The interaction of RRT use on the association of BAR with in-hospital mortality was also significant (*P* = 0.006) (Figure 3).

#### Sensitivity analysis

After excluding missing data from the full cohort (*n* = 13,464) (Supplementary Table 1), 9,081 patients were left and the relationship between BAR and in-hospital mortality remained reliable (HR: 1.09, 95% CI: 1.07–1.12, *P* < 0.001). After excluding patients of ICU stay < 24 h and < 48 h, this relationship remained stable, as shown in Supplementary Table 2 (HR: 1.09, 95% CI: 1.06–1.12, *P* < 0.001) and Supplementary Table 3 (HR: 1.08, 95% CI: 1.05–1.11, *P* < 0.001). In addition, the same results were observed in the population of AKI (HR: 1.09, 95% CI: 1.06–1.11, *P* < 0.001) (Supplementary Table 4).

## Discussion

The present study demonstrated that BAR was independently associated with poor outcomes in ICU patients with sepsis. Elevated BAR levels were significantly associated with lower survival rates in patients with sepsis.

Previous literature has demonstrated that BUN is a reliable prognostic biomarker of sepsis (17–19, 38), and albumin is negatively correlated with sepsis (39). BUN is one of the redundant outcomes of protein metabolism in the liver, while blood urea is filtered out through the glomerulus and undergoes renal tubular reabsorption (14). The protein catabolism rate in patients with sepsis is significantly increased, and it is often complicated by acute kidney injury (40). Therefore, patients with sepsis have elevated foundational BUN levels (17, 18), and sepsis can significantly reduce blood flow and renal function, which further increases BUN levels (41). In septic AKI, evidence suggests that inflammatory mediators, immune cell infiltration, and nitric oxide synthase dysregulation lead to reduced renal blood flow in the renal parenchyma, resulting in independent microcirculatory dysfunction (42). BUN is influenced by some factors related to patient conditions (39). In contrast, malnutrition status may reduce plasma albumin levels, while systemic inflammatory responses have the same effect (15, 16). Hypoalbuminemia indicates the severity of inflammation and can be an additional risk stratification biomarker for mortality and prognosis, with an acute drop in serum albumin levels immediately after infection predicting a poor prognosis (37). In addition, hepatic dysfunction, kidney damage, and other conditions can reduce serum albumin levels (43, 44). Therefore, previous scholars introduced some albumin-based ratios for the diagnosis and prognosis of sepsis (7, 45).

The causes and pathophysiological mechanisms underlying the relationship between BAR and adverse prognosis remain unclear. BAR can be evaluated as a comprehensive body reserve by considering the four conditions of malnutrition, dehydration, liver reserve, and kidney reserve and may be more useful than BUN or serum albumin in assessing disease severity (21). Furthermore, BAR is simpler and easier to calculate, is not affected by individual subjectivity, and is more convenient for clinical use. Many studies have illustrated that a higher BAR level is related to the relative deficiency of effective circulating blood volume, suggesting that BAR can be a useful tool to guide volume management in patients with sepsis (46). Some researchers have studied the relationship between BAR and the prognosis of patients with respiratory diseases. Ryu et al. showed that BAR is a useful prognostic factor for aspiration pneumonia (47). Similar results were reported by Fang et al. in a prognostic study of patients with critical acute pulmonary embolism (24). In addition, a retrospective analysis of 602 patients with coronavirus-19 concluded that elevated BAR at admission was an independent predictor of mortality during hospitalization in patients with COVID-19 (25). Prognostic studies of BAR and other diseases, such as chronic heart failure (46) and *Escherichia coli* bacteremia (48), have also been reported. The possible mechanisms of the high BAR and poor prognosis of sepsis can be explained in terms of BUN and albumin levels. A high BAR represents a high BUN concentration and a low albumin level. Renal hypoperfusion and hypoalbuminemia often occur in critically ill patients, leading to increased BUN and BAR levels. This will help therapists understand the implications of elevated BAR levels and evaluate the prognosis of patients.

However, previous studies had limited sample sizes. This study, using the MIMIC-IV database, which by far included the largest population, proved that BAR could be a biomarker correlated with mortality. In our study, we retrospectively enrolled 13,464 patients with sepsis using the large public MIMIC-IV database and proved that the initial BAR was associated with in-hospital mortality in patients with sepsis. This association remained stable even after multivariate analysis and subgroup analysis that were used to eliminate the imbalance and confounding factors of covariates across the BAR groups. An interactive effect between age and BAR on sepsis outcome was found; that is, BAR was significantly and positively associated with the risk of in-hospital mortality in older participants (≥65 years). A possible reason for this is that the BAR of older people is sensitive to variations. This result suggests that BAR may be a more helpful predictor that strengthens clinicians’ capacity to access prognosis for elderly patients with sepsis, consequently improving management strategies. Additionally, there were potential interactions between RRT use on the association between BAR and in-hospital mortality. However, we are cautious about this result because of unequal sample sizes. Therefore, the exact mechanism underlying this finding needs to be explored in future studies.

Our study has several advantages. First, the study used real-world data from a large and diverse population. Second, this was a retrospective observational study, which was susceptible to potential confounding factors. Strict statistical adjustments were used to minimize residual confounders. We considered the target independent variables as both continuous and categorical variables. With this approach, the contingency in the data analysis was reduced, and the robustness of the results was enhanced. Moreover, the effect modifier factor analysis improved the usage of the data and yielded more robust results in different subgroups. Finally, to our knowledge, this is the first study to observe an independent association between in-hospital mortality and sepsis in intensive care patients. The findings of this study will be helpful for future research in establishing diagnostic and predictive models for in-hospital mortality.

However, this retrospective study had some limitations. First, the values of serum albumin or BUN may change over time, and this study only included the initial BAR value without monitoring its dynamic change, although these values may be more accurate in predicting the prognosis of sepsis. Second, we discarded many variables extracted from the database due to missing data. These variables included serum C-reactive protein, PCT levels, and some other inflammation-related biomarkers, which may be helpful for researchers to elucidate the mechanism of sepsis mortality. Third, for patients with multiple ICU admissions, only the first ICU admission was included in the analysis, which may have generated a selection bias. In addition, BUN levels may be influenced by diet. However, we did not obtain dietary information about the participants because of the limitations of the MIMIC-IV database. Finally, this retrospective study was based on the MIMIC-IV database. Although efforts have been made to minimize confusion caused by confounding factors, there are still some potential confounding factors that have not been determined. Further high-quality prospective multicenter studies are necessary to validate the prognostic value of BAR for sepsis and investigate the underlying mechanisms.

In this study, we identified the relationship between the initial BAR value and in-hospital mortality in ICU patients with sepsis. Elevated BAR was significantly associated with lower survival in patients with sepsis in the ICU in the USA.

## Data availability statement

The raw data supporting the conclusions of this article will be made available by the authors, without undue reservation.

## Ethics statement

Ethical review and approval was not required for the study on human participants in accordance with the local legislation and institutional requirements. Written informed consent for participation was not required for this study in accordance with the national legislation and the institutional requirements.

## Author contributions

SC and QW designed the study. CC, CG, and LZ conducted the data collection and data analysis. SC wrote the manuscript. MY modified the manuscript. All authors contributed to the article and approved the submitted version.

## Footnotes

1
http://www.R-project.org
