# Supplementary material for: Association between blood urea nitrogen to serum albumin ratio and in-hospital mortality of patients with sepsis in intensive care: A retrospective analysis of the fourth-generation Medical Information Mart for Intensive Care database
Source: Front Nutr. 2022 Nov 4;9:967332. doi: 10.3389/fnut.2022.967332 (PMC9672517; doi:10.3389/fnut.2022.967332)
Supplement: Supplementary file 1 [file Table_1.DOCX]

**TABLE S1** | Sensitivity analysis of patients after excluded missing data from the study

| Variable | n | Unadjusted | |  | Model 1 | | Model 2 | | Model 3 | |
| --- | --- | --- | --- | --- | --- | --- | --- | --- | --- | --- |
|  |  | HR 95CI% | *P* value |  | HR 95CI% | *P* value | HR 95CI% | *P* value | HR 95CI% | *P* value |
| BAR^a^ | 9081 | 1.14 (1.12~1.16) | <0.001 |  | 1.13 (1.12~1.15) | <0.001 | 1.139 (1.112~1.167) | <0.001 | 1.09 (1.07~1.1) | <0.001 |
| BAR4 |  |  |  |  |  |  |  |  |  |  |
| Q1(BAR<4.85) | 2069 | 1(Ref) |  |  | 1(Ref) |  | 1(Ref) |  | 1(Ref) |  |
| Q2(4.85≤BAR<7.86) | 2254 | 1.41 (1.12~1.66) | <0.001 |  | 1.27 (1.08~1.5) | 0.004 | 1.1 (0.93~1.3) | 0.253 | 1 (0.84~1.12) | 0.978 |
| Q3(7.86≤BAR<13.9) | 2341 | 2.16 (1.85~2.51) | <0.001 |  | 1.847 (1.58~2.16) | <0.001 | 1.37 (1.17~1.61) | <0.001 | 1.08 (0.91~1.27) | 0.380 |
| Q4(BAR ≥13.9) | 2417 | 3.02 (2.61~3.48) | <0.001 |  | 2.585 (2.23~3) | <0.001 | 2.006 (1.7~2.38) | <0.001 | 1.43 (1.2~1.71) | <0.001 |
| *P* for trend |  |  | <0.001 |  |  | <0.001 |  | <0.001 |  | <0.001 |

Abbreviation: BAR, Blood urea nitrogen to serum albumin ratio;

^a^ BAR was entered as a continuous variable per 5 unit

Model 1 = Adjusted for (age+gender)

Model 2 = Model1+(ethnicity+HR+MAP+SpO_2_+hemoglobin+SCr+platelets+WBC+chloride+glucose+lactate+pH)

Model 3 = Model 2+(weight+malignant cancer+severe liver disease+renal disease+CCI+APSIII+SOFA score+urine output+ventilator use+RRT use+vasopressin usage).
