# Supplementary material for: Association between blood urea nitrogen to serum albumin ratio and in-hospital mortality of patients with sepsis in intensive care: A retrospective analysis of the fourth-generation Medical Information Mart for Intensive Care database
Source: Front Nutr. 2022 Nov 4;9:967332. doi: 10.3389/fnut.2022.967332 (PMC9672517; doi:10.3389/fnut.2022.967332)
Supplement: Supplementary file 2 [file Table_2.DOCX]

**TABLE S2** | Sensitivity analysis of excluding patients with ICU stay < 24 h

| Variable | n | Unadjusted | |  | Model 1 | | Model 2 | | Model 3 | |
| --- | --- | --- | --- | --- | --- | --- | --- | --- | --- | --- |
|  |  | HR 95CI% | *P* value |  | HR 95CI% | *P* value | HR 95CI% | *P* value | HR 95CI% | *P* value |
| BAR^a^ | 13228 | 1.14 (1.12~1.15) | <0.001 |  | 1.12 (1.11~1.14) | <0.001 | 1.14 (1.11~1.17) | <0.001 | 1.09 (1.06~1.12) | <0.001 |
| BAR4 |  |  |  |  |  |  |  |  |  |  |
| Q1(BAR<4.85) | 3323 | 1(Ref) |  |  | 1(Ref) |  | 1(Ref) |  | 1(Ref) |  |
| Q2(4.85≤BAR<7.86) | 3342 | 1.42 (1.22~1.64) | <0.001 |  | 1.27 (1.09~1.47) | 0.002 | 1.18 (1~1.4) | 0.050 | 1.07 (0.9~1.27) | 0.460 |
| Q3(7.86≤BAR<13.9) | 3310 | 2.15 (1.87~2.46) | <0.001 |  | 1.84 (1.6~2.12) | <0.001 | 1.5 (1.27~1.77) | <0.001 | 1.16 (0.98~1.38) | 0.081 |
| Q4(BAR ≥13.9) | 3253 | 2.87 (2.52~3.27) | <0.001 |  | 2.47 (2.16~2.83) | <0.001 | 2.23 (1.88~2.66) | <0.001 | 1.52 (1.27~1.82) | <0.001 |
| *P* for trend |  |  | <0.001 |  |  | <0.001 |  | <0.001 |  | <0.001 |

Abbreviation: BAR, Blood urea nitrogen to serum albumin ratio;

^a^ BAR was entered as a continuous variable per 5 unit

Model 1 = Adjusted for (age+gender)

Model 2 = Model1+(ethnicity+HR+MAP+SpO_2_+hemoglobin+SCr+platelets+WBC+chloride+glucose+lactate+pH)

Model 3 = Model 2+(weight+malignant cancer+severe liver disease+renal disease+CCI+APSIII+SOFA score+urine output+ventilator use+RRT use+vasopressin usage)
