# Supplementary material for: Association between blood urea nitrogen to serum albumin ratio and in-hospital mortality of patients with sepsis in intensive care: A retrospective analysis of the fourth-generation Medical Information Mart for Intensive Care database
Source: Front Nutr. 2022 Nov 4;9:967332. doi: 10.3389/fnut.2022.967332 (PMC9672517; doi:10.3389/fnut.2022.967332)
Supplement: Supplementary file 3 [file Table_3.DOCX]

**TABLE S3** | Sensitivity analysis of excluding patients with ICU stay < 48 h

| Variable | n | Unadjusted | |  | Model 1 | | Model 2 | | Model 3 | |
| --- | --- | --- | --- | --- | --- | --- | --- | --- | --- | --- |
|  |  | HR 95CI% | *P* value |  | HR 95CI% | *P* value | HR 95CI% | *P* value | HR 95CI% | *P* value |
| BAR^a^ | 12884 | 1.13 (1.11~1.15) | <0.001 |  | 1.11 (1.09~1.13) | <0.001 | 1.13 (1.1~1.16) | <0.001 | 1.08 (1.05~1.11) | <0.001 |
| BAR4 |  |  |  |  |  |  |  |  |  |  |
| Q1(BAR<4.85) | 3245 | 1(Ref) |  |  | 1(Ref) |  | 1(Ref) |  | 1(Ref) |  |
| Q2(4.85≤BAR<7.86) | 3278 | 1.43 (1.23~1.67) | <0.001 |  | 1.27 (1.09~1.48) | 0.002 | 1.23 (1.03~1.47) | 0.024 | 1.09 (0.91~1.3) | 0.360 |
| Q3(7.86≤BAR<13.9) | 3225 | 2.13 (1.85~2.46) | <0.001 |  | 1.81 (1.56~2.09) | <0.001 | 1.61 (1.35~1.92) | <0.001 | 1.2 (1~1.43) | 0.050 |
| Q4(BAR ≥13.9) | 3136 | 2.76 (2.4~3.16) | <0.001 |  | 2.35 (2.04~2.7) | <0.001 | 2.3 (1.92~2.77) | <0.001 | 1.47 (1.21~1.79) | <0.001 |
| *P* for trend |  |  | <0.001 |  |  | <0.001 |  | <0.001 |  | <0.001 |

Abbreviation: BAR, Blood urea nitrogen to serum albumin ratio;

^a^ BAR was entered as a continuous variable per 5 unit

Model 1 = Adjusted for (age+gender)

Model 2 = Model1+(ethnicity+HR+MAP+SpO_2_+hemoglobin+SCr+platelets+WBC+chloride+glucose+lactate+pH)

Model 3 = Model 2+(weight+malignant cancer+severe liver disease+renal disease+CCI+APSIII+SOFA score+urine output+ventilator use+RRT use+vasopressin usage)
