# Supplementary material for: Association between blood urea nitrogen to serum albumin ratio and in-hospital mortality of patients with sepsis in intensive care: A retrospective analysis of the fourth-generation Medical Information Mart for Intensive Care database
Source: Front Nutr. 2022 Nov 4;9:967332. doi: 10.3389/fnut.2022.967332 (PMC9672517; doi:10.3389/fnut.2022.967332)
Supplement: Supplementary file 4 [file Table_4.DOCX]

**TABLE S4 |** Sensitivity analysis of patients with AKI

| Variable | n | Unadjusted | |  | Model 1 | | Model 2 | | Model 3 | |
| --- | --- | --- | --- | --- | --- | --- | --- | --- | --- | --- |
|  |  | HR 95CI% | *P* value |  | HR 95CI% | *P* value | HR 95CI% | *P* value | HR 95CI% | *P* value |
| BAR^a^ | 7667 | 1.13 (1.11~1.15) | <0.001 |  | 1.13 (1.11~1.15) | <0.001 | 1.14 (1.11~1.17) | <0.001 | 1.09 (1.06~1.11) | <0.001 |
| BAR4 |  |  |  |  |  |  |  |  |  |  |
| Q1(BAR<4.85) | 1304 | 1(Ref) |  |  | 1(Ref) |  | 1(Ref) |  | 1(Ref) |  |
| Q2(4.85≤BAR<7.86) | 1866 | 1.33 (1.1~1.61) | 0.003 |  | 1.24 (1.02~1.5) | 0.028 | 1.11 (0.91~1.34) | 0.293 | 1.01 (0.83~1.22) | 0.955 |
| Q3(7.86≤BAR<13.9) | 2173 | 2.01 (1.68~2.4) | <0.001 |  | 1.81 (1.52~2.17) | <0.001 | 1.4 (1.16~1.68) | <0.001 | 1.09 (0.9~1.31) | 0.381 |
| Q4(BAR ≥13.9) | 2324 | 2.85 (2.4~3.38) | <0.001 |  | 2.59 (2.18~3.08) | <0.001 | 2.08 (1.72~2.52) | <0.001 | 1.45 (1.19~1.76) | <0.001 |
| *P* for trend |  |  | <0.001 |  |  | <0.001 |  | <0.001 |  | <0.001 |

Abbreviation: BAR, Blood urea nitrogen to serum albumin ratio;

^a^ BAR was entered as a continuous variable per 5 unit

Model 1 = Adjusted for (age+gender)

Model 2 = Model1+(ethnicity+HR+MAP+SpO_2_+hemoglobin+SCr+platelets+WBC+chloride+glucose+lactate+pH)

Model 3 = Model 2+(weight+malignant cancer+severe liver disease+renal disease+CCI+APSIII+SOFA score+urine output+ventilator use+RRT use+vasopressin usage)
